# Attentional biases in abstinent patients with cocaine use disorder: rapid orienting or delayed disengagement?

**DOI:** 10.3389/fpsyg.2024.1290890

**Published:** 2024-01-31

**Authors:** Victoria Branchadell, Rosario Poy, Pilar Segarra, Pablo Ribes-Guardiola, Javier Moltó

**Affiliations:** Affective Neuroscience Lab, Department of Basic and Clinical Psychology, and Psychobiology, Universitat Jaume I, Castelló, Spain

**Keywords:** attentional bias, cocaine use disorder, abstinence, dot-probe task, attentional disengagement

## Abstract

Addiction-related attentional biases may play a central role in the development and maintenance of drug-seeking and drug-taking behaviors. However, evidence in cocaine dependence is limited and mixed. This study examined the time course and component processes of attentional biases for cocaine-related cues in a sample of 47 outpatients (38 men) with cocaine use disorder (CUD) with varying durations of current abstinence. Reaction times in a visual dot-probe task with two picture exposure durations —500 ms, to assess initial stages of attention, and 2,000 ms, to assess maintained attention— were recorded. We found faster responses to probes replacing cocaine-related vs. matched control pictures in the 500 ms but not in the 2,000 ms condition, indicative of early but not late attentional biases for cocaine cues in abstinent patients with CUD. Further comparisons with a neutral baseline revealed that it was not due to rapid orienting but to delayed disengagement from cocaine-related pictures, being this effect greater the longer the period of current abstinence. Consistent with the incentive-sensitization theory, these data suggest that cocaine-related stimuli maintain the capacity to hold spatial attention in abstinent patients with CUD, even after months of abstinence, highlighting the relevance of carrying out stimulus control to avoid relapses.

## 1 Introduction

Several explanations of drug dependence have proposed that attentional biases —defined here as the tendency of substance-related cues to capture the attention of substance abusers (Field and Cox, 2008)— may play a central role in the development and maintenance of drug-seeking and drug-taking behaviors. According to the incentive-sensitization theory (Robinson and Berridge, 1993, 2008), the chronic use of potentially addictive substances can persistently sensitize brain structures that regulate the attribution of unconscious incentive salience to drug and drug-related stimuli (mesocorticolimbic dopamine systems; for a recent revision of evidence supporting a hyperdopaminergic reactivity state in addiction; see Samaha et al., 2021), being this process the main responsible for drug-seeking and drug-taking behavior, by means of transforming the psychological representations of those stimuli so that they “grab attention” and become especially wanted, thus eliciting approach behavior to the goal. The mechanism proposed by the incentive-sensitization theory may be also relevant for behavioral addictions, with the stimuli acquiring incentive salience due to repeated neuroadaptations by the frequent involvement in the addictive behavior [e.g., see in gambling disorder: (Ciccarelli et al., 2016; Kim et al., 2021); for a systematic review: (Hønsi et al., 2013); or in internet addiction: (Cannito et al., 2023)].

The visual dot-probe and the Stroop tasks have been the most widely used measures to study addiction-related attentional biases, with the dot-probe task providing a more direct and ecologically valid assessment of attentional orienting toward or away from drug-related cues (Robbins and Ehrman, 2004). On each trial of this procedure, a pair of pictures appears side by side on the screen —being one member of the pair a drug-related picture and the other member a matched, neutral picture. When the two pictures disappear, a probe stimulus is presented in the location of one of them. Participants are instructed to make a forced-choice response indicating the probe identity, orientation, or location. Attentional bias to drug-cues is indicated by faster reaction times to probes replacing drug-related stimuli than to probes replacing matched neutral stimuli —based on the assumption that responses are typically faster to stimuli that appear in attended locations of the visual field (see Posner et al., 1980).

Another advantage of the dot-probe task is that this procedure allows to examine whether biases operate in early or later stages of the attentional process (*cf.* LaBerge, 1995), by simply manipulating the picture exposure duration. Attentional effects at short exposure durations (usually, 200 ms or less) are thought to measure fast, automatic biases in the initial orienting of attention *toward* highly salient stimuli, whereas attentional effects at longer exposure durations (1,000 ms or more) are assumed to reflect controlled, continued maintenance of attention *on* substance-related cues (see, for example, Field and Cox, 2008; Frankland et al., 2016). Attentional effects at intermediate exposure durations (such as the widely used 500 ms) are interpreted as reflecting either facilitated initial orienting —given its positive correlation with direction of first eye movements to significant stimuli (Bradley et al., 2000)— or delayed disengagement *from* salient stimuli —as indexed by slower responses to probes replacing matched neutral stimuli compared to a neutral baseline (Koster et al., 2005). A 500-ms exposure duration thus provides the opportunity for examining specific components underlying attentional biases (facilitated engagement vs. delayed disengagement), which could aid to clarify the role of biased attention for drug-related cues in the maintenance of addiction (and in the vulnerability to relapse following treatment; Marhe et al., 2013; Garland and Howard, 2014).

Attentional biases for pictorial drug cues using the dot-probe task have been extensively examined in smokers and alcohol users (Robbins and Ehrman, 2004; Field and Cox, 2008), but studies in cocaine dependence are scarce and have yielded mixed evidence. On the one hand, research has failed to demonstrate attentional biases in active cocaine users for pictorial drug cues presented for 200 ms (Mayer et al., 2016), for 500 ms (Montgomery et al., 2010; see also Mayer et al., 2016, reporting interference for cocaine stimuli) or for 1,000 ms (Marks et al., 2014, 2015).^1^ On the other hand, a preliminary study in our laboratory on the time course of attentional biases in 16 abstinent patients with cocaine use disorder (CUD) found that these individuals (compared to non-user controls) showed attentional biases for cocaine-related pictures presented for 500 ms, but not for 2,000 ms (Ventura et al., 2011), a finding that seems to suggest that the bias operates in earlier stages of the attentional process and not in later stages of controlled, maintained attention. The question of whether these mixed results are due to variations in the experimental procedure, to small sample sizes, or, most importantly, to differences in the cocaine use status (active users vs. abstinent outpatients) remains unanswered.

Relatedly, it would be helpful to clarify if attentional biases persist after drug consumption cessation (and, if so, for how long). Findings from studies exploring this question using the dot-probe task are not consistent across substance abuse disorders, with some studies reporting significantly lesser automatic attentional bias in former users than in current opiate addicts (Constantinou et al., 2010) and frequent ketamine users (Morgan et al., 2008), and others reporting similar attentional biases in former and current smokers (Rehme et al., 2018). To our knowledge, there are no studies in participants with CUD aimed at testing directly for the relationship between duration of abstinence and level of attentional bias in the dot-probe task. Given its possible implications in relapses, this might be an important factor to consider.

Thus, the aim of the present study was to examine, in a larger and more heterogeneous sample of abstinent CUD patients than the one in Ventura et al. (2011), the time course and component processes —orienting, disengagement, and/or maintenance of attention— of attentional biases for cocaine-related cues, also exploring the potential role of abstinence duration. To this end, a mixed-gender sample of CUD patients undergoing psychological treatment, with differing periods of abstinence, performed a visual dot-probe task that included two picture exposure durations: a 500 ms condition to assess facilitated orienting of attention to, or delayed disengagement from, cocaine-related cues, and a 2,000 ms condition to assess maintained attention to these stimuli. The usual comparison between responses to probes replacing addiction-related vs. matched control stimuli was supplemented, when necessary, by comparing these reaction times on trials containing cocaine-related pictures with reaction times on trials containing only neutral pictures, in order to differentiate attentional capture by cocaine cues (faster responding to probes replacing cocaine pictures than on neutral-pair trials) and disengagement difficulties from this material (slower responding to probes replacing matched control pictures than on neutral-pair trials; *cf.* Koster et al., 2004). Based on preliminary evidence (Ventura et al., 2011), we expected to find biases in the early stages of the attentional process but not in maintained attention to cocaine-related pictures. As regarding component processes of the hypothesized attentional bias (orienting or disengagement), no specific hypotheses were posited.

## 2 Materials and methods

### 2.1 Participants

Fifty-four^2^ patients with cocaine use disorder (CUD) —according to DSM-5 (American Psychiatric Association, 2013) criteria— in outpatient psychological treatment in a center for addictive behaviors participated in the present study. None of the participants met criteria for mental disorders other than CUD. All participants had been abstinent for 3 to 459 days (*M* = 102.4, *SD* = 104.7) at the time of the study. Abstinence was measured by self-report and verified by weekly urine analyses since the beginning of treatment. This study was approved by the Ethical Committee of the University and complies with ethical principles for human research set in the Declaration of Helsinki. All participants were informed about the nature of the study, provided informed consent, and were compensated for their time.

Seven participants were excluded because of extreme outlying response times on the task (see *Data reduction and statistical analyses* section). The final sample comprised a total of 47 participants (38 men) between the ages of 20 and 51 (*M* = 32.7, *SD* = 7.8), 38.3% of whom were in early or sustained remission according to DSM-5 specifiers. Sociodemographic characteristics and drug use measures for the experimental sample are presented in Table 1. None of the sociodemographic, cocaine use, or craving variables showed a significant correlation with the duration (in days) of current abstinence (*r*s < |0.26|; *p*s > 0.10).

**Table 1:** Sociodemographic characteristics and drug use measures for the overall sample (*n* = 47).

Measure	Mean (Standard Deviation)	Range
Gender (male/female)	38/9	
Studies (primary education or less/at least secondary education)^a^	18/23	
Currently employed/Unemployed^a^	18/23	
Married/Single^a^	11/30	
Daily consumption over the 6 months prior to treatment (no/yes)^a^	26/15	
Previous treatments (no/yes)^a^	26/15	
Age at onset of cocaine use (years)^a^	20.0 (5.1)	13–34
Duration of use (years)^b^	8.7 (6.7)	1–29
Cocaine Craving Questionnaire-General Brief^c^	50.0 (10.5)	30–67
Drug Desire Questionnaire^c^	24.8 (12.9)	13–73
Abstinence	102.4 (104.7)	3–459

Superscripts letters designate group sizes (^a^*n* = 41; ^b^*n* = 38; ^c^n = 46).

### 2.2 Materials

The visual dot-probe task and stimuli were the same as in Ventura et al. (2011). The stimuli in critical trials consisted of 20 color images of cocaine-related scenes (e.g., a CD with two lines of cocaine on it) and 20 corresponding control pictures matched as closely as possible for content and perceptual characteristics, but lacking any cocaine-related cues (e.g., a CD with two parallel stickers). Forty additional neutral pictures (household objects) without cocaine cues were selected from the *International Affective Picture System*^3^ (IAPS; Lang et al., 2008) based on Spanish normative ratings on valence and arousal (Moltó et al., 1999, 2013; Vila et al., 2001) to be used in 20 randomly generated neutral-pair trials. The fact that cocaine cues did not appear on every trial allowed us to obtain a neutral baseline to test whether the hypothesized attentional effects in critical trials reflected facilitated orienting to cocaine cues or delayed disengagement from them (see Koster et al., 2004, 2005). Each picture was 8 cm wide and 6 cm high when presented side by side on the screen, with a distance of 11 cm between the centers of the pictures in each pair. The task was programmed and presented using the Presentation v9.2 software (Neurobehavioral Systems, Inc. Albany, CA) on a PC HP workstation xw4600 computer with a 22” LCD monitor. A Cedrus RB-730 response box was employed to record responses with 1 ms accuracy. The overall duration of the task was approximately 10 min.

### 2.3 Procedure

The procedure was as described in Ventura et al. (2011). Each participant was contacted at the addictive behaviors center. After an informed consent was obtained, the experimenter collected sociodemographic information and history of drug use data through a semi-structured interview and the review of clinical files, and the participant completed a general craving measure (Cocaine Craving Questionnaire-General Brief; CCQ; Tiffany et al., 1993). The experimental session took place within 2 to 4 weeks later in a research laboratory. Participants completed an instant cocaine craving measure (Drug Desire Questionnaire; DDQ; Franken et al., 2002) before beginning the task. Participants performed the visual dot-probe task in a soundproof and dimly room seated approximately at 1.5 m of the screen monitor where the stimuli were displayed. Before starting the task, an experimenter entered into the room and read out loud the task instructions, which were also projected on the screen in front of the participant. Each trial started with a central fixation cross (“+”) for 1,000 ms, followed by a pair of pictures arranged horizontally and displayed for either 500 or 2,000 ms. The picture pair was replaced with a dot (probe) that appeared on either the left or right side of the screen in the spatial location where one of the pictures had been. Participants were instructed to press —with the corresponding index finger— one of two assigned buttons as quickly as possible, while avoiding mistakes, to indicate the location of the probe (left, right). The task began with 14 practice trials followed by 240 trials (160 critical and 80 neutral trials), divided into two equal blocks with a 1-min rest break in the middle. On critical trials, each cocaine-related picture appeared on either the left or right side of the screen with equal frequency, and the probe replaced each cocaine-related picture and its matched control picture an equal number of times in each spatial location. On neutral-pair trials, probes appeared on the left or right with equal frequency. Trials were presented in random order for each participant.

### 2.4 Data reduction and statistical analyses

Reaction times (RTs) to probes (in ms) from trials with incorrect responses were removed (1.35%), as well as RTs greater than or less than two standard deviations from the participant’s mean (3.31%). Mean RT to probes on critical trials was calculated for each participant, separately for each cueing condition (probe replaces cocaine-related pictures vs. control pictures) and picture duration (500 ms, 2,000 ms); mean RT to probes on neutral trials was calculated for each participant separately for each picture duration. Seven participants (2 women) were excluded entirely from further analyses due to extreme outlying mean RTs (> 2 *SD*s) in any of the six experimental conditions. RT data for all conditions were normally distributed (all Shapiro–Wilk’s W > 0.96, *p*s > 0.11) and showed good split-half reliability (odd-even method, Spearman-Brown corrected): for the 500 ms condition, coefficients for probes replacing cocaine-related, control, and neutral pictures were 0.98, 0.97, and 0.94, respectively; for the 2,000 ms condition, the corresponding values were 0.95, 0.97, and 0.96.

Attentional bias effects in the overall sample were evaluated by a 2 (Cueing Condition: cocaine, control) × 2 (Picture Duration: 500 ms, 2,000 ms) repeated measures analysis of variance (ANOVA) in which both main and interaction effects were tested; the Greenhouse–Geisser correction was applied when appropriate. The neutral trial type was not included in this analysis because it cannot be assigned to either of two competing cueing conditions (cocaine vs. control). Significant cueing effects were deeply evaluated by comparing RTs on critical trials with RTs on neutral-pair trials, to clarify whether potential attentional biases reflected facilitated orienting *toward* cocaine pictures (faster RTs to probes replacing cocaine pictures compared to neutral ones in neutral trials) or delayed disengagement *from* these cues (slower RTs to probes replacing control pictures compared to neutral ones in neutral trials; *cf.* Koster et al., 2004). Further, in order to test for the unique predictive contribution of abstinence duration on component processes of significant attentional biases for cocaine cues, hierarchical regression analyses were conducted for each RT variable of interest. In these analyses, RTs to probes replacing either cocaine-related or matched control pictures for the picture exposure condition of interest served as the criterion, and gender (men = 0, women = 1), age, and RTs to probes replacing neutral pictures for the corresponding picture exposure condition were included as predictors in Step 1, followed by craving measures (i.e., CCQ and DDQ scores) in Step 2, and abstinence duration (in days) in Step 3.

## 3 Results

Table 2 shows mean RTs to probes as a function of cueing condition and picture duration. Participants responded faster to probes replacing pictures displayed for 2,000 ms than to those displayed for 500 ms (415 vs. 437 ms, *Picture Duration F*_1,46_ = 52.00, *p* < 0.001, *η*_p_^2^ = 0.53), and to probes replacing cocaine pictures than to those replacing control pictures (425 vs. 428 ms, *Cueing Condition F*_1,46_ = 5.26, *p* = 0.026, *η*_p_^2^ = 0.10). Interestingly, participants responded faster to probes replacing cocaine vs. control pictures in the 500 ms condition (434 vs. 441 ms, respectively, *p* = 0.003), but not in the 2,000 ms condition (415 vs. 415 ms, *p* = 0.95) (*Cueing Condition x Picture Duration* interaction *F*_1,46_ = 6.01; *p* = 0.018, *η*_p_^2^ = 0.12), thus providing evidence of early but not late attentional biases for cocaine-related cues in abstinent patients with CUD.

**Table 2:** Mean (SD) reaction times (ms) to probes replacing cocaine-related and matched control pictures (critical trials) and neutral pictures (neutral-neutral trials) as a function of picture duration (500 ms, 2,000 ms) in the overall sample (*n* = 47).

	Cueing condition		Neutral trials
Picture duration	Cocaine pictures	Control pictures	Neutral pictures
500 ms	434 (53)	441 (52)	435 (54)
2,000 ms	415 (51)	415 (51)	407 (47)

**Figure 1:**
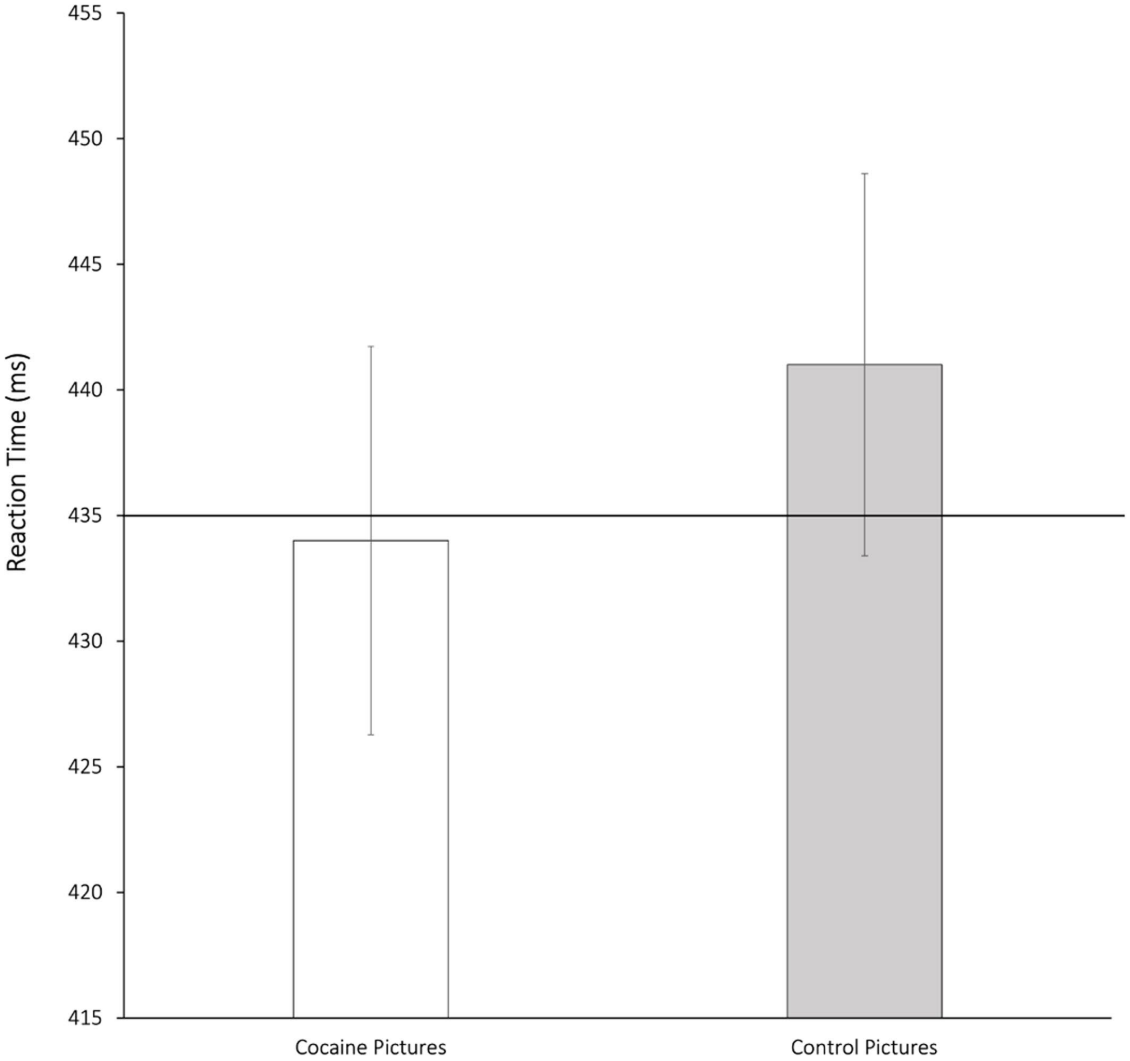
Mean reaction times (ms) and standard errors to probes replacing 500 ms-presented cocaine-related pictures and matched control pictures compared to reaction times on neutral-neutral trails (horizontal line) in the overall sample (*n* = 47).

The nature of this significant attentional effect was pursued by conducting paired-samples *t*-tests between responses on critical and neutral-pair trials in the 500 ms condition: compared to probes replacing neutral pictures (*M* = 435 ms, *SD* = 54), participants responded significantly slower to probes replacing control pictures (*t*_46_ = −2.27, *p* = 0.028, *d* = 0.11), and displayed comparable reaction times to probes replacing cocaine pictures (*p* = 0.74), indicating that the early attentional bias found in abstinent patients with CUD was not due to rapid orienting but to delayed disengagement from cocaine-related cues. Figure 1 illustrates this finding. As there was no evidence of significant cueing effects in the 2,000 ms condition, further comparisons with responses on the neutral baseline were not justified (*cf.* Koster et al., 2005).

**Figure 2:**
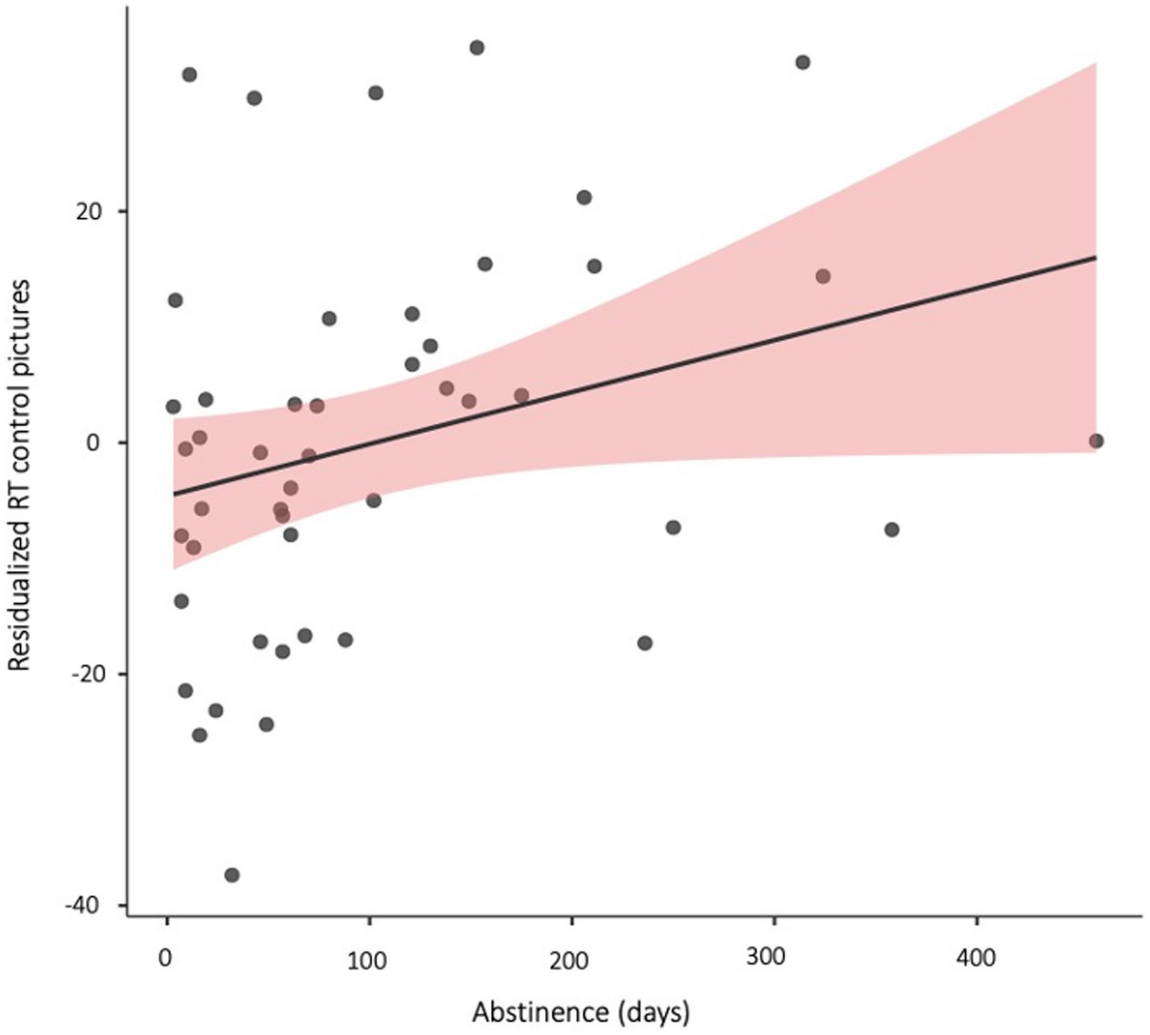
Scatterplot depicting the correlation between abstinence duration (days) and residualized reaction times to probes replacing 500 ms-presented matched control pictures (controlling for age, gender, and reaction times on neutral-neutral trails).

The subsequent hierarchical regression analysis revealed that abstinence duration contributed significantly (*β* = 0.10) to the prediction of slower RTs to probes replacing 500 ms-presented control pictures (*F*_1, 39_ = 4.12, *p* = 0.049, Δ*R*^2^ = 0.008), after controlling for age, gender, RTs on neutral trials in the 500 ms condition, and CCQ and DDQ scores (see coefficients in Table 3). This finding reflected greater difficulties to disengage attention from cocaine-related pictures for participants with longer durations of abstinence (see Figure 2).

**Table 3:** Standardized β weights from hierarchical regression predicting reaction times (ms) to probes replacing 500 ms-presented matched control pictures.

Step 1	β_1_	*R*^2^ = 0.899***
Age	−0.08	
Gender	0.07	
RTs Neutral Pictures	0.94***	

Step 2	β_2_	𝚫*R*^2^ = 0.012
Age	−0.06	
Gender	0.05	
RTs Neutral Pictures	0.92***	
CCQ	0.07	
DDQ	0.07	

Step 3	β_3_	𝚫*R*^2^ = 0.008*
Age	−0.08	
Gender	0.04	
RTs Neutral Pictures	0.96***	
CCQ	0.05	
DDQ	0.08	
Abstinence	0.10*	

β_**x**_, standardized β for each predictor in the model at Step x; Δ*R*^2^, increase in explained variance at Step 2 and Step 3; CCQ, Cocaine Craving Questionnaire-General Brief (Tiffany et al., 1993); DDQ = Drug Desire Questionnaire (Franken et al., 2002).

Significant associations are highlighted in bold.

*p < 0.05, **p < 0.01, ***p < 0.001.

## 4 Discussion

Our study provides novel findings on component processes of biases in visual orienting in abstaining individuals with cocaine use disorder, with potential implications for the prevention of relapse. We found a significant attentional bias for the 500 ms but not for the 2,000 ms condition in the whole sample of abstinent CUD patients that reflected delayed disengagement from cocaine-related pictures rather than facilitated orienting to these cues. Importantly, the magnitude of these disengagement difficulties increased with the duration of current abstinence. These results are discussed in more detail below.

Our results in a sample of abstinent patients with CUD suggest that biases for cocaine-related cues operate in early, more involuntary stages of the attentional process, as hypothesized, rather than in later, controlled stages of maintained attention. This finding extends to a larger and more heterogeneous sample of patients with CUD the results of Ventura et al.’s (2011) study in an identical visual dot-probe procedure, and is highly consistent with data about the time course of attentional biases in other substance abuse disorders, such as smoking (Bradley et al., 2003; Rehme et al., 2018) and opioid dependence (Garland et al., 2013; Frankland et al., 2016). Importantly, these same studies have consistently reported no attentional biases in the voluntary maintenance of attention to drug-related stimuli, as assessed in 1,500 and 2,000 ms exposure conditions in the visual dot-probe task (but see Bradley et al., 2003). Evidences suggesting that initial shiftings of attention preferentially lead processing resources to drug-related cues are consonant with the idea that stimuli with high incentive salience for the individual acquire the capacity to automatically capture attention (Robinson and Berridge, 1993).

Findings from a deeper examination of the specific components involved in the initial orienting of attention were of particular relevance: as compared to a neutral baseline, participants in our study showed slower responses when the probe replaced matched control pictures but not faster responses when it replaced cocaine-related pictures. This pattern of results suggests that attentional biases in abstinent patients with CUD might be driven by a difficulty to *disengage attention from* cocaine-related cues rather than by a facilitated *orienting to* these stimuli [see (Heitmann et al., 2020), for corresponding evidence for alcohol-related cues, and (Field and Cox, 2008), for interpretation of attentional biases with exposure durations of 500 ms]. Evidence in cocaine users for larger fixation times on cocaine-related pictures (see Marks et al., 2014, 2015) and for longer response times and increased occipital activity for task-irrelevant cocaine-related stimuli (Hester and Garavan, 2009) would be also consistent with this explanation.

A further contribution of this study was the finding that delayed disengagement from cocaine-related stimuli varied with the duration of current abstinence, with participants with longer abstinence periods showing greater attentional effects. The fact that CUD patients with shorter abstinence periods had less difficulty in disengaging attention resembles previous results failing to show attentional biases for pictorial drug cues in active cocaine users (e.g., Montgomery et al., 2010; Mayer et al., 2016). One possible explanation may be that participants in our study with shorter duration of current abstinence had been exposed to the substance itself more recently —for example, patients with CUD that were not in remission (61.7% of our sample) were abstinent, on average, 38 days (*SD* = 26.6). In contrast, deprivation of cocaine use for longer periods might have sensitized the reward value of substance-related cues —i.e., increasing incentive salience (Robinson and Berridge, 1993)— and thus enhanced attentional capture by cocaine-pictures in these participants —consistent with EEG/ERP research on incubation of cue-induced craving in abstinent individuals with CUD (Parvaz et al., 2016; see also Parvaz et al., 2017) and on decreased attentional bias in current (vs. abstinent) cocaine users (Dunning et al., 2011). Cocaine-seeking behavior following extinction has also been demonstrated to increase with the duration of cocaine unavailability in rats (Di Ciano and Everitt, 2002). This explanation is supported by findings of early attentional biases in the dot-probe task in smokers with repeated quit attempts but not in those without a history of repeated quitting (Bradley et al., 2003), as well as of stronger attentional bias in the Stroop task in treatment-seeking (as compared to non-treatment-seeking) cocaine-dependent individuals (Vadhan et al., 2007). The differential magnitudes of attentional bias for CUD patients depending on the duration of abstinence might be likely explained by the time elapsed since the last consumption of cocaine, but also perhaps by disorder severity, or treatment adherence, among other variables, so further research is needed to address this question.

Evidence about a greater difficulty to disengage attention from cocaine-related cues in abstinent patients with CUD may have important implications for treatment. Difficulties in shifting the attention away from cocaine cues in abstinent cocaine abuse patients have been related to craving, obsessive thoughts, and experienced control over cocaine use (Franken et al., 2000) —with important potential effects on the probability of relapse. Further, attentional bias modification has not proved to be more effective than a control therapy at reducing attentional bias or craving in cocaine users (Mayer et al., 2016, 2020), and our results may help inform interventions that directly modify the specific component of visual orienting in need of change (i.e., disengagement). Thus, consistent with evidence suggesting that the capacity of cocaine-related stimuli to hold visual attention is inversely related to prefrontal cortex-mediated cognitive control (Hester and Garavan, 2009), the introduction of cognitive reappraisal (a self-regulation strategy that activates domain-general cognitive control regions in the prefrontal cortex; Buhle et al., 2014) in attentional bias modification has been shown to be effective in reducing spontaneous attentional bias to cocaine-related pictures in individuals with CUD (Parvaz et al., 2021). In parallel, our findings also highlight the importance of carrying out stimulus control in all treatments for abstinence in cocaine use disorder (for a specific explanation, see Bickel and Kelly, 1988). Since attentional biases for drug-related stimuli seem to persist for a long time even after drug consumption cessation, relapses will likely occur if the individual does not avoid contact with these stimuli.

One of the main limitations is that the present study did not have a non-users control group *per se*, although the methodological validity of the current experimental procedure had already been demonstrated in a preliminary study (Ventura et al., 2011) using the same visual dot-probe task and stimuli. Furthermore, our sample was composed mostly by men. Although gender was not found to be a significant predictor of attentional biases in our study, these results should be replicated in gender-balanced samples. Moreover, future studies could consider using more sensitive methods than urine analyses to verify abstinence, such as hair tests. It would have been also interesting to incorporate shorter exposure conditions (200 ms or less), in order to unequivocally index automatic engagement of attention. Additionally, and although widely used as an index of attentional bias in the dot-probe task, reaction time measurements could be complemented in future research with other measures that have proven more sensitive to assess attentional bias for cocaine-related cues, such as fixation times and electrophysiological measures (Rosenberg, 2009; Marks et al., 2014, 2015).

In conclusion, our study showed delayed disengagement of attention from cocaine-related pictures —rather than initial facilitated orienting to these cues— in abstinent patients with CUD. This finding holds relevance for the understanding of the nature of attentional biases for cocaine stimuli in abstinent patients —as further research might help to differentiate the implications of engagement and disengagement biases in the maintenance of substance use disorders— and, importantly, for the optimization of interventions by targeting the specific component of visual orienting for therapeutic change.

## Data availability statement

The raw data supporting the conclusions of this article will be made available by the authors, without undue reservation.

## Ethics statement

The studies involving humans were approved by Human Research Ethics Committee of the Universitat Jaume I. The studies were conducted in accordance with the local legislation and institutional requirements. The participants provided their written informed consent to participate in this study.

## Author contributions

VB: Data curation, Formal analysis, Investigation, Writing – original draft, Writing – review & editing. RP: Conceptualization, Formal analysis, Methodology, Software, Writing – original draft, Writing – review & editing. PS: Conceptualization, Methodology, Software, Writing – review & editing. PR-G: Data curation, Investigation, Writing – review & editing. JM: Conceptualization, Funding acquisition, Methodology, Project administration, Software, Supervision, Writing – review & editing.

## Footnotes

^1^Two other studies in cocaine-dependent samples using the dot-probe task with 500 ms-presented pictures (see Tull et al., 2011; Bardeen et al., 2014) did not report analyses on attentional bias at baseline —focusing instead in between groups comparisons of attentional bias scores after intervention—, and thus they could not be reviewed here. A similar rationale was applicable to Fernández-Calderón et al.’s (2021) study, which only reported the relationships between attentional bias scores for 200 ms and 1,000 ms-presented pictures and cocaine use disorder severity of patients in treatment.

^2^A sensitivity power analyses conducted in G*Power (see Faul et al., 2007) revealed that a repeated measures ANOVA (within factors) with 47 participants across four conditions would be sensitive to effects of *η_p_^2^* = 0.03 with 80% power (α = 0.05).

^3^The IAPS identification numbers for the 40 neutral pictures used in the neutral-pair trials were: 7000, 7001, 7003, 7004, 7006, 7010, 7011, 7014, 7017, 7018, 7019, 7020, 7021, 7025, 7030, 7042, 7053, 7055, 7056, 7057, 7059, 7061, 7079, 7080, 7095, 7096, 7100, 7136, 7150, 7175, 7190, 7192, 7207, 7211, 7217, 7224, 7233, 7235, 7705, 9401.
